# Liability of Operators and Anæsthetists

**Published:** 1908-01-11

**Authors:** 


### LIABILITY OF OPERATORS AND ANÆSTHETISTS.

Under the above title the *Law Journal* of December 14 discusses the question of the legal liability to prosecution for *mala praxis* or for manslaughter of operating surgeons and anæsthetists in cases of deaths during operations. The question is one on which there has, happily, so far been no leading case, and in consequence it is one on which there is much doubt and uncertainty. The *Law Journal* thinks that, at an inquest held by the Southwark Coroner on December 7, besides this it was also established that "anæsthetics . . . on occasion . . . are administered . . . by newly-fledged practitioners whose knowledge of the effect of drugs is by no means equal to that of an ordinary chemist." It deserves to be put on record that the latter half of this assertion cannot for one moment be admitted, nor can it be legitimately deduced from the evidence at the inquest referred to. The inference drawn is much less open to objection, namely, that patients who submit to operation in hospitals "are entitled to some guarantee that the utmost skill and care shall be used." Even that position, however, seems to be in advance of the usual statement of the text-books that the practitioner does not undertake to use the highest possible skill, but only a fair, reasonable, and competent degree thereof.

A more important point than this, however, is raised in regard to the apportionment of responsibility between operator and anæsthetist. The *Law Journal* thinks individual cases must be decided on their merits, which is a most reasonable attitude. The laying down of any hard and fast rule on the subject is undesirable, because circumstances differ so very widely in different cases, but one or two broad principles certainly seem to require the attention of the profession. It has been suggested more than once that the operator should be held liable for the whole of the operation, including the administration of the anæsthetic; but the majority of operating surgeons would, no doubt, decline to accept this latter responsibility at all. There are, however, some surgeons, and of the utmost eminence, whose constant supervision of their anæsthetists might justify the latter in declining any legal liability. Again, patients are very commonly anæsthetised in a room adjoining that in which the operation is to take place; the induction period is that in which a very considerable proportion of all fatalities occurs: to make, therefore, the surgeon responsible for events at which he is not present seems too much opposed to reason to be acceptable to a jury.

In the inquest under consideration the prospective operator was asked whether he took any responsibility for the administration, and he quite rightly negatived the idea; the anæsthetist, when questioned, accepted the responsibility in full. When the operator himself chooses his anæsthetist it might conceivably be argued that he should be made responsible for the consequences, though that seems most unjust provided the anæsthetist is of fair average competence; but when the operator and anæsthetist are associated by virtue of a roster of duty in an institution where they are both in office, the proposition is surely untenable.

There are other cases in which the relative position is obscured by various factors. Thus, at another recent inquest the surgeon told the Court that he had been "keeping half an eye" on the anæsthetist, who was admittedly a practitioner of small experience. Had the jury found culpable neglect in that particular case, the surgeon might have found himself in the uncomfortable position of prisoner on a charge of manslaughter, and very possibly the anæsthetist too. Yet another difficult situation may arise when an anæsthetist, as occasionally happens, warns a surgeon that their patient is doing badly during an operation. In such circumstances, the surgeon sometimes decides to continue in spite of the risk, and if his conduct were afterwards called in question he would doubtless be required to justify the proceeding as necessary in the interests of the patient. In such a case it is clear that no responsibility ought to attach to the anæsthetist, even though death may reasonably be ascribed to the action of the drug used. In extreme instances such as these the distribution of responsibility and liability is easily made: it is in mishaps occurring suddenly in an ordinary surgical operation that doubt and difficulty may arise owing to the lack of any clear definition of the exact legal status of the surgeon and the anæsthetist. To ascertain the general consensus of opinion in the profession on the point might be difficult, but would not be impossible to such a body as the Royal Society of Medicine; it would be of very great service to medicine and to law if some pronouncement on this important question could be obtained.

